# Supplementary material for: High syphilis prevalence and incidence in people living with HIV and Preexposure Prophylaxis users: A retrospective review in the French Dat’AIDS cohort
Source: PLoS One. 2022 May 19;17(5):e0268670. doi: 10.1371/journal.pone.0268670 (PMC9119478; doi:10.1371/journal.pone.0268670)
Supplement: Attachment — Submitted filename: Response to Reviewers.docx [file pone.0268670.s003.docx]

Thomas Lemmet

Nouvel Hôpital Civil, Quai Louis Pasteur

Strasbourg

March 31^th^, 2022

Subject: Rebuttal letter, PONE-D-21-37034

Title: High syphilis prevalence and incidence in people living with HIV and Preexposure Prophylaxis users: a retrospective review in the French Dat’AIDS cohort

Authors: Thomas LEMMET, Laurent COTTE, Clotilde ALLAVENA, Thomas HULEUX, Claudine DUVIVIER, Hélène LAROCHE, André CABIE, Pascal PUGLIESE, Thomas JOVELIN, Marine MAUREL, Cyrille DELPIERRE, David REY

Dear Editors,

The authors would like to thank the reviewers for their helpful comments on the manuscript. Please find the edited manuscript linked to this letter. The manuscript has been improved according to the suggestions of the reviewers:

Regarding the academic editor remarks:

1. We added a minimal data sets regarding PLWH and PrEP users as Supporting Informations with this new submission. Theses datas can be used by interested researchers to replicate our findings in their entirety.
2. The members of the Dat’AIDS Study Group are listed at the end of the manuscript, and Email address of the leading author of each center is now provided.
3. Figures were checked with the Preflight Analysis and Conversion Engine (PACE) digital diagnostic tool.

Regarding the reviewers remarks:

1. The reference concerning the public health data from the French organization “Santé publique France” was added.
2. We did not dispose of data regarding sexual partners of PrEP users. However, we assumed they were mostly men, as we know most of PrEP users are MSM.
3. Since comparing PrEP users and people living with HIV was not our aim, the design of the study was unfit to do so. Therefore, we decided not to compare the two groups.
4. Concerning the discussion regarding PrEP users, we added some references regarding data available about syphilis in this population.
5. Counseling to reduce sexual risks, and to analyze behavior increasing exposure to STIs, was part of routine clinical assessment in PLWH and PrEP users, as well as identification of chemsex consumption, specifically in PrEP users.
6. In case of acute syphilis diagnosis, antibiotic treatment was prescribed according to recommendations, usually benzathine penicillin (in absence of allergy); however, we didn’t extract this item in the database, fearing underreporting in medical computer files; same comment for neurosyphilis, which are in addition very rare, thus without additional value.
7. For statistical analysis, the variables chosen for the multivariate model were selected from the literature as factors potentially associated with the risk of syphilis infection. Besides, the underlying assumptions of the multivariate logistic regression were tested and the residuals analysed. No violations were found and the goodness of fit of the model was good.

Last but not least, we made some adjustments throughout this new version of the manuscript
in order to correct the layout and some turns of phrases. Numbers and acronyms were uniformed, and we also proceed to check the grammar. The discussion part also benefited from a reorganization so as not to anticipate the limits of our study.

We hope that our modifications render our manuscript in its current form suitable for publication in Plos One.

Best regards,

On behalf of the authors,

Thomas Lemmet
